# Fatigue, muscle fatigability, and the Hallmarks of Aging: a narrative review

**DOI:** 10.1016/j.jnha.2026.100844

**Published:** 2026-04-09

**Authors:** Anna Ronca, Federico Bellelli, Marco Proietti, Yves Rolland, Bruno Vellas, Philipe de Souto Barreto

**Affiliations:** a IHU Health Age, Toulouse, France; b Division of Subacute Care, IRCCS Istituti Clinici Scientifici Maugeri, Milan, Italy; c Institut du Vieillissement, Gérontopole of the Centre Hospitalo-Universitaire of Toulouse, Toulouse, France; d Department of Clinical Sciences and Community Health, University of Milan, Milan, Italy; e Division of Cardiogeriatric Subacute Care, IRCCS Istituti Clinici Scientifici Maugeri, Milan, Italy; f CERPOP, UMR 1295, Université Paul Sabatier, Toulouse, France

**Keywords:** Fatigue, Hallmarks of ageing, Geroscience, Human study

## Abstract

**Background:**

Fatigue is a common symptom in the general population, and its prevalence increases with advancing age, suggesting that it may represent an age-related condition. However, the underlying pathophysiological mechanisms of fatigue during aging remain poorly understood, limiting the identification of potential therapeutic targets. This review summarises current evidence on the associations between the main biological drivers of ageing and both self-perceived fatigue and muscle fatigability.

**Methods:**

Broad search terms related to fatigue and the hallmarks of ageing were combined. Studies using any study design were included if they involved human participants and reported associations between fatigue and biomarkers of ageing. Studies were excluded if they focused solely on patients with specific diseases or if fatigue was embedded within specific symptom clusters.

**Results:**

Thirty studies were included; they were published between 2005 and 2024. Most studies (83%) involved community-dwelling subjects; 63% were cross-sectional. Mitochondrial dysfunction and chronic inflammation showed the strongest associations with self-perceived fatigue and, to a lesser extent, muscle fatigability. Mitochondrial dysfunction may contribute via impaired electron transport and early anaerobic metabolism, while inflammatory markers (e.g., interleukin-6, C-reactive protein) may act via central nervous system pathways and persistent subclinical systemic inflammation. We found no evidence linking epigenetic alterations, proteostasis dysfunction, and impaired macroautophagy to fatigue. Other hallmarks show limited evidence.

**Conclusions:**

Despite its clinical relevance, the interplay between the biological drivers of ageing and the fatigue is still poorly understood, although current findings suggest a role for mitochondrial dysfunction and chronic inflammation. Further investigations may help identify potential pathways for treating fatigue.

## Highlights

• Fatigue increases with age, but its biological causes are unclear, hindering effective treatment development.
• Mitochondrial dysfunction and chronic inflammation are key ageing drivers linked to fatigue and muscle fatigability.
• Other hallmarks of ageing show little evidence, underscoring the need for more research on fatigue mechanisms and therapies.

## 1 Introduction

Fatigue is a common health complaint among older adults, having a significant impact on individuals’ quality of life [1]. It is typically defined as the feeling of weakness, lack of energy and tiredness [2], and it is frequently associated with a series of adverse outcomes, including functional limitations, disability, hospitalisation and death [3]. Although fatigue is often attributed to systemic diseases or other identifiable causes - such as COVID-19, cancer or multiple sclerosis [4] - it may occur in the absence of a clear underlying condition [5]. Beyond its prevalence, there remains no consensus on how to define and operationalise fatigue in older adulthood [2].

The *Geroscience* hypothesis posits that ageing, as a biological process, is closely linked to the development of chronic diseases, functional decline in older individuals, and other age-related conditions [6]. *Lopez-Otín* et al. (2023) identified twelve cellular and molecular pathways driving the ageing process, the so-called “*Hallmarks of Ageing*”. Fatigue is increasingly recognized as a multidimensional syndrome rather than a unidimensional symptom [7]. This understanding has led to the development of different approach for its assessment, including questionnaires that capture the self-perception of fatigue in daily life and physical performance measures aimed at eliciting or quantifying the presence of the symptom [8]. Subjective fatigue may influence performance, and task-induced muscle fatigability may contribute to the perception of fatigue. Since self-reported fatigue as well as muscle fatigability increase with age [3,9], they may constitute age-related conditions. Moreover, fatigue is highly prevalent in age-associated conditions such as cardiovascular disease, sarcopenia, frailty, metabolic dysfunction, and neurodegenerative disorders [1,10], all of which share key biological mechanisms of ageing. This convergence of clinical and mechanistic evidence supports the view that ageing processes may contribute causally to fatigue, rather than it being solely a secondary feature of specific diseases.

Despite that, no study has synthesised current evidence on how the biological mechanisms of fatigue and ageing intersect. Understanding how fatigue relates to the hallmarks of ageing could help clarify its pathophysiology, identify potential biomarkers, and inform targeted interventions.

The aim of this narrative review is to gather the current evidence from human studies on how the hallmarks of ageing related to both self-perceived fatigue and muscle fatigability during aging.

## 2 Methods

Although a systematic review was out of the scope of the present work, in order to avoid losing important information in this field, we searched for articles on the biological aspects of fatigue through the perspective of hallmarks of ageing in the *PubMed* database in September 2025. No restrictions were placed on the publications’ year, language of screened studies, or study design. Broad search terms, defined on the basis of prior literature [8,11], related to fatigue (e.g., fatigue, tiredness) and biological ageing (the 12 hallmarks of ageing) were combined using AND combinations (see the search strategy in Supplementary Material Table-S1). Articles were included if they met all inclusion criteria and none of the exclusion criteria. Inclusion criteria were: (i) studies conducted in cohorts of adult humans (≥18 years) and (ii) studies investigating the association between fatigue (self-reported fatigue and/or muscle fatigability) and a biological mechanism corresponding to one of the 12 hallmarks of ageing. Exclusion criteria were defined on both clinical and formal grounds. From clinical perspective, studies were excluded when fatigue was examined exclusively within a broader clinical conditions, such as COVID-19 infection and autoimmune diseases, or where fatigue was embedded within specific symptom constellations (e.g., cancer-related or chemotherapy-related fatigue [12], multiple sclerosis–related fatigue [13], or myalgic encephalomyelitis/chronic fatigue syndrome [14], according to the specific diagnostic criteria of each entity). From a formal perspective, meta-analyses, review articles and editorials were excluded. For the purposes of the present review, the term “fatigue” refers to global self-reported symptoms, whereas the concept of muscle fatigability refers to the degree of fatigue experienced during performance of a defined activity [15]. Since it is not known if the pathophysiology of these conditions is similar or not, we opted to discuss findings on fatigue and muscle fatigability separately hereafter. When a study provided information about either self-perceived fatigue and muscle fatigability, it was included in both sections. We conceptually organised the molecular and cellular drivers of aging according to their hypothesised roles within the overall biological ageing processes - primary drivers, antagonistic responses and integrative markers [11].

## 3 Results and discussion

Among 161 manuscripts selected for full-text evaluation, 30 studies were included in this review. The eligibility flow diagram is presented in Supplementary Material Table-S1. Included studies were published between 2005 and 2024. Fourteen of them were conducted in Europe, twelve in United States and only four elsewhere. Most of the studies (83%) included only community-dwelling individuals and almost two out of three (63%) had a cross-sectional design. Table 1 summarises the characteristics of each study.

**Table 1:** Summary of findings.

First Author, year	Study Design	Populations Characteristic	Type of Fatigue	Hallmarks of aging
Bradley E. et al., 2009	Longitudinal (4 months), weekly follow up	n = 168 oncology outpatients with breast, prostate, lung or brain cancer undergoing RT treatment (AA 60.9 ± 11.6 years, 44,6% F); n = 85 healthy controls from patients' family caregivers (AA 62.5 ± 10.5 years, 71,8% F) in California	Perceived Fatigue	Genomic Instability
Rain Katz et al., 2022	Longitudinal study from a ongoing population study on aging, enrolment and first visit in 2006/09, annual telephone follow-up, second visit in 2014/17	n = 1997 community-dwelling older adults in United States and Denmark (AA 73.7 ± 10.4 years, 54,4% F)	Perceived Fatigue	Telomere attrition
Lafortuna C.L. et al., 2013	CS	n = 635 outpatients aged 19−78 years (54.9% F) with obesity class II and III from Italy consecutively admitted to hospital for treatment of severe adiposity excess	Perceived Fatigue	Deregulated Nutrient Sensing
Kurajoh M. et al., 2016	CS, patients of Hyogo Sleep Cardio-Autonomic Atherosclerosis Study	n = 347 community-dwelling patients with 1 or more cardiovascular risk factors (AA 59,5 ± 12,7 years, 53,6% M)	Perceived Fatigue	Deregulated Nutrient Sensing
Stringer E. A. et al., 2013	Longitudinal (25 consecutive days of blood draws and self-reporting of symptom severity)	n = 10 chronic fatigue syndrome patients (AA 52,9 years) and n = 10 healthy controls (AA 53,0 years)	Perceived Fatigue	Deregulated Nutrient Sensing
Herpich C. et al., 2021	CS	n = 45 hospitalized geriatric patients without CI or cancer, from Berlin. n = 22 fatigued patients (AA 74.9 ± 8.4 years, 43.5% F); n = 23 non fatigued patients (AA 77.8 ± 5.8 years, 45.5 F)	Perceived Fatigue	Mitochondrial dysfunction
Liu F. et al., 2020	Longitudinal; Sample from ongoing Baltimore Longitudinal Study of Aging (BLSA); different follow-up intervals depending on age: every 4 years for age <60, every 2 years for age 60–79, and every year for age ≥80.	n = 544 community dwelling older adults free of major chronic conditions and CI at the time of enrollment (AA 70 ± 10 years, 56.5% F)	Perceived Fatigue	Mitochondrial dysfunction
Wawrzyniak N. R. et al., 2016	CS	n = 48 community dwelling older adults from Florida, without conditions overtly related to fatigue symptoms. n = 20 patients with idiopathic chronic fatigue (AA 72.4 ± 5.9 years, 45% F); n = 28 non fatigued patients (AA 72.4 ± 4.9 years, 48,2% F)	Perceived Fatigue	Mitochondrial dysfunction
Yuichiro O. et al., 2024	CS	n = 22 healthy male participants from Japan; n = 13 young group (AA 32.9 ± 6.7 years); n = 9 elderly group (AA: 55.1 ± 6.0 years)	Muscle Fatigability	Cellular Senescence
Soendenbroe C. et al., 2022	Longitudinal (7 days); 3 visits for fatigue assessment	Community-dwelling Danish adult men; n = 15 young controls (AA: 26 ± 5 years); n = 16 elderly practicing life-long recreational exercise (AA: 73 ± 4 years); n = 15 age-matched sedentary controls (AA: 73 ± 4 years)	Muscle Fatigability	Stem Cell Exhaustion
Alway S. E. et al., 2017	CT	Community-dwelling older individuals in West Virginia; 12 weeks resveratrol treatment group = 15 (AA: 67.9 ± 1.1 years; 60% F); age and sex-matched controls n = 15	Muscle Fatigability	Stem Cell Exhaustion
Capuron L. et al., 2011	CS; subsample from 7-year follow up of Three-City (3C) epidemiological cohort study	n = 284 community-dwelling older adults living in Bordeaux, Dijon and Montpellier (AA 79.9 ± 4.5 years; 69,4% F)	Perceived Fatigue	Altered Intercellular communication
Bautmans I. et al., 2007	CS; from a longitudinal survey	n = 40 community-dwelling older adults from Brussels (AA 75.5 ± 5.5 years; 37.5% F)	Perceived Fatigue and Muscle Fatigability	Chronic Inflammation
Bautmans I. et al., 2005	Comparative Study	n = 63 hospitalized geriatric patients in Brussels (AA 84.2 ± 5.7 years; 66.7% F) divided in two groups according to C-reactive protein and fibrinogen blood concentrations	Muscle Fatigability	Chronic Inflammation
Bautmans I. et al., 2008	CS	n = 77 residents from 3 nursing homes in Brussels (AA 81 ± 8 years, 74.6% F)	Perceived Fatigue and Muscle Fatigability	Chronic Inflammation
Cooper R. et al., 2017	Longitudinal; from National Survey in Scotland with regular life-long follow-up; 24th data collection in 2014/15; postal questionnaire at age 68; nurse home visit at age of 69	n = 1580 community dwelling older adults who undergo a clinical and biochemical assessment at age 60-64 and, then, physical activity assessment at age of 68 (49.4% women)	Perceived Fatigue	Chronic Inflammation
Feng L. et al., 2014	Observational laboratory stress reactivity study	n = 55 community-dwelling individuals (AA 82.95, 56.4% F) from a local community senior center in Northeastern U.S. city	Perceived Fatigue	Chronic Inflammation
Nicklas B. J. et al., 2016	Longitudinal (18 months); subsample from the Intensive Diet and Exercise for Arthritis Study	n = 167 obese community-dwelling middle-aged and older adults (AA 66.2 ± 6.4, 70% F), 3 groups: diet, exercise and combination of diet and exercise	Perceived Fatigue	Chronic Inflammation
Silva J. P. et al., 2010	CS	n = 135 sedentary community elderly women (AA 71.2 ± 4.57) from Brazil	Perceived Fatigue and Muscle Fatigability	Chronic Inflammation
Sorensen J. R., et al., 2018	Comparativd study of 3 days	n = 19 community-dwelling participants from U.S.A.; n = 11 sedentary or mildly active young adults (AA 22 ± 2 years, 36,4% F); n = 8 older adults able to complete the physical task (AA 71 ± 7 years, 0% F)	Muscle fatigability	Chronic Inflammation
Valentine R. J. et al., 2011	CS	n = 182 healthy community-dwelling older adults (AA 69,2 ± 6,7, 46,2% F)	Perceived Fatigue	Chronic Inflammation
Wanigatunga A. A. et al., 2018	Sample from ongoing Baltimore Longitudinal Study of aging (BLSA) on human aging; different follow-up intervals depending on age: every 4 years for age <60, every 2 years for age 60–79, and every year for age ≥80.	n = 985 participants (baseline AA 70 ± 10 years, 53% F) divided into 2 groups based on interleukine-6 serum levels (cut off 3,7 pg/mL)	Perceived Fatigue	Chronic Inflammation
Hoekstra C. et al., 2023	CS. from Geriatric Resilience Registry in Radboud University Medical Centre (Netherlands) for hospitalized geriatric patients	n = 104 hospitalized geriatric patients (AA 83,3 ± 7,5, 55,8% F) with a median number of 6 chronic conditions	Perceived Fatigue	Chronic Inflammation
Hughes A. et al.; 2017	Longitudinal analysis from UKHLS, an annual longitudinal survey of a general population sample of 40000 English households (annual interviews and blood samples from a single nurse visit after 2/3 annually interviews)	n = 10606 community-dwelling English adults divided into 3 groups based on the fatigue assessment: low fatigue (AA 50.6 ± 16.3, 51,2% F), mid fatigue (AA 51,7 ± 17,1, 57,3% F) and high fatigue (AA 54,3 ± 17,4,63,3% F)	Perceived Fatigue	Chronic Inflammation
Arnold P. et al., 2017	CS, hospitalized patients from Universitair Zirkenhuis Brussels and community-dwelling older controls from senior associations	n = 10 hospitalized geriatric patients with acute inflammation (AA 82,0 ± 5,7, 80% F) and n = 19 older controls (AA 75,5 ± 6,2, 47% F)	Muscle fatigability	Chronic Inflammation
White J. et al., 2016	CS from the English Longitudinal Study of Aging	n = 5909 (AA 66 years; 54.9% F)	Perceived Fatigue	Chronic Inflammation
Avlund K. et al., 2012	Comparative study recruiting from 9 general practitioners in Denmark	n = 196 young individuals aged 20−35 and n = 314 older individuals aged 70−85	Perceived Fatigue	Chronic Inflammation
Vestergaard S. et al., 2009	CS from a population-based sample	n = 1055 community-dwelling old Italian adult (AA 74,5 ± 7,0, 56,0% F)	Perceived Fatigue	Chronic Inflammation
Valentine R. J. et al., 2009	CS	n = 127 community-dwelling older adults (F = 80, AA 69,5 ± 5,4; M = 47, AA 70,6 ± 5,4)	Perceived Fatigue	Chronic Inflammation
Zang Q. et al., 2022	CS	n = 36 patients with major depressive disorders (AA 36.81 ± 13.52, 41,6% F) after hospital admission; n = 45 controls from nearby communities (AA 39.29 ± 11.44, 57,8% F) in Beijing	Perceived Fatigue	Dysbiosis

Legend: n = number of patients in the sample, CS = cross-sectional, CT = clinical trial, AA = average age, F = females, M = males CI = cognitive impairment.

### 3.1 Perceived fatigue and hallmarks of ageing

We found 24 articles investigating the relationship between self-perceived fatigue and at least one hallmark of ageing. A summary of findings from studies on self-perceived fatigue is provided in Table 2.

**Table 2:** Summary of findings from studies on perceived fatigue.

First author, year	Fatigue assessment	Hallmark of Aging	Hallmark of Aging assessment	Investigation results
Bradley E., 2009	Lee Fatigue Scale (LFS): mean of 13 items ranging from 0 to 10, higher scores indicate higher levels of fatigue	Genomic instability	Blood sample; whole genome amplification from archived buffy coat specimens	TNFα-308 minor allele was associated with lower levels of morning fatigue and lower levels of symptom severity longitudinally
Rain Katz, 2022	Pittsburgh Fatigability Scale (PFS): sum of 10 items assessing perceived fatigue (0−5), higher scores indicate greater fatigue, cut point of ≥15 establish greater perceived fatigue	Telomere attrition	Blood sample; LTL from real-time PCR with primers optimized for telomeres after DNA extraction	Shorter LTL predicts higher perceived fatigue at 8 years; LLT was associated with perceived fatigue in the older
Lafortuna C.L. et al., 2013	Fatigue Severity Scale (FSS): 9-items measuring fatigue through its effect on daily functioning, higher scores indicate greater fatigue	Deregulated Nutrient Sensing	Standard anthropometric measurements at hospital admission (including body composition assessment); fasting blood sample (fasting plasma glucose, fasting serum insulin).	Fatigue was correlated with body mass excess, glucose metabolism impairment (fasting plasma glucose and insulin) and poor motor performance, especially in women
Kurajoh M. et al., 2016	Fatigue Score Severity: 64-items that assess how often patients experienced fatigue-associated symptoms in the last week, maximum score is 20 which indicates higher levels of fatigue	Deregulated Nutrient Sensing	Fasting blood sample; leptin concentrations were measured using enzyme-linked immunosorbent assay kit	Plasma leptin concentrations in a model that includes age was associated with moderately fatigued conditions
Stringer E. A. et al., 2013	Fatigue Severity: Visual analogue scale (VAS) asking, “Overall, how severe has your fatigue been today?”. The far left of the scale was anchored at “no fatigue” and the far right was anchored at “severe fatigue”	Deregulated Nutrient Sensing	Blood sample	One healthy control demonstrated significant positive correlation between fatigue and leptin; no associations were found between fatigue and the 51 cytokines in the control group
Herpich C. et al., 2021	Brief Fatigue Inventory (BFI): first question asking if the patient feels more fatigued than usual; 3 questions asking for the intensity of fatigue; and 6 questions asking for the impairment in daily living. Items on severity and impairment range from 0 to 10, higher scores correspond to higher fatigue	Mitochondrial dysfunction	Fasting blood sample; peripheral blood mononuclear cells were freshly isolated and centrifuged; cellular oxygen consumption analysis from cells	Age-related fatigue was associated with reduced mitochondrial function in peripheral blood mononuclear cells; Fatigue was associated with lower OCR and lower adenosine tri-phosphate ATP turnover.
Liu F. et al., 2020	Borg Rating of Perceived Exertion (RPE) scale after a treadmill walk of 5 min: range 6−20. A higher score indicates higher perceived fatigability.	Mitochondrial dysfunction	Indirect calorimetry for respiratory exchange ratio	Age-related fatigability was inversely associated with mitochondrial function and earlier utilization of anaerobic metabolism
Wawrzyniak N. R. et al., 2016	FACIT Fatigue Scale (FACIT-F): 13 self-reported items that relate to fatigue during daily activities over the past 7 days rating on a 4-point scale; higher total scores correspond to higher fatigue	Mitochondrial dysfunction	Biopsies of skeletal muscle samples from the vastus lateralis; total OXPHOS antibody cocktail for electron transport chain subunit proteins identification	In fatigue individuals there were reduction of cytochrome C oxidase, AMPK activation, lower levels of mitochondrially-localized Sirtuine 3 protein, alterations of complex IV and V of the electron transport chain
Capuron L. et al., 2011	Multidimensional Fatigue Inventory (MFI): 20-item questionnaire assessing 5 dimensions of fatigue ranging from 4 (best) to 20 (worst)	Altered Intercellular Communication	Fasting blood sample for serum inflammatory markers and amino acids measurement	Chronic low-grade inflammation in aging was associated with increased tryptophan catabolism and altered phenylalanine turnover; potential pathophysiological mechanisms of fatigue in elderly people
Bautmans I. et al., 2007	Visual Analogue Scale for Fatigue (VAS-F) score from 0−10 (higher scores higher fatigue) and Mobility-Tiredness Scale (Mob-T) for fatigue following daily-life activities (high scores less fatigue)	Chronic Inflammation	Blood sample for CRP and IL-6	No association between self-perceived fatigue and IL-6 levels
Bautmans I. et al., 2008	Visual Analogue Scale for Fatigue (VAS-F) score from 0−10 (higher scores higher fatigue) and Mobility-Tiredness Scale (Mob-T) for fatigue following daily-life activities (high scores less fatigue)	Chronic Inflammation	Blood sample for IL-6, TNF-α and Hsp70	Self-reported fatigue was significantly related with muscle endurance; Worse muscle endurance was related to higher levels in both IL-6 and Hsp70
Cooper R. et al., 2017	Pittsburgh Fatigability Scale (PFS): sum of 10 items rating perceived fatigue (0−5), higher scores indicate greater fatigue, cut point of ≥15 establish greater perceived fatigue	Chronic Inflammation	Overnight fasting blood samples and anthropometric measures taken by nurses at age 60–64	BMI and IL-6 at age 60–64 were associated with higher fatigability at age 68; underweight or obese participants and those with higher levels of IL-6 experienced higher perceived fatigue than those of normal weight and with lower levels of IL-6, respectively
Lin F. et al., 2014	Multidimensional Fatigue Inventory (MFI): 20-item self-rating measuring five dimensions of fatigue (general fatigue, physical fatigue, mental fatigue, reduced activity and reduced motivation) ranging from 4 (best) to 20 (worst)	Chronic Inflammation	Capillary blood sample for assaying chemokines and cytokines	The fatigability cluster showed higher levels of IL-6 and greater IL-6 reactivity both at the baseline and after cognitive stimulation compared to individuals with low fatigability levels
Nicklas B. J. et al., 2016	Vitality domain on the Medical Outcomes 36-item short-form (SF-36) measure: the vitality subscale consists in 4 items assessing fatigue in the past month rating from 1 to 6; scores are linearly transformed to a scale of 0−100 with higher scores indicating less fatigue	Chronic Inflammation	Fasting blood samples	Fatigue did not correlate with CRP or IL-6 at the baseline; larger decline in both CRP and IL-6 associated with lower fatigue scores
Silva J. P. et al., 2010	Visual Analogue Scale for fatigue (VAS-F): with graduation in colours, ranging from light blue (minimal) to intense red (maximal), and a numerical scale ranging from 0 to 10, which quantified the level of fatigue reported	Chronic Inflammation	Blood sample for plasma concentrations of the inflammatory mediators IL-6 and soluble receptor of TNFα	No association between self-perceived fatigue and IL-6 levels
Valentine R. J. et al., 2011	Multidimensional Fatigue Inventory (MFI): 20-item self-rating measuring five dimensions of fatigue (general fatigue, physical fatigue, mental fatigue, reduced activity and reduced motivation) ranging from 4 (best) to 20 (worst)	Chronic Inflammation	Blood sample after overnight fasting and 5−10 min rest	Adiposity was positively associated with fatigue, with the exception of mental fatigue; CRP, IL-6 and WBC count were related to several dimensions of fatigue; adiposity independently explained a significant amount of the variance in general and physical fatigue
Wanigatunga A. A. et al., 2018	Borg Rating of Perceived Exertion (RPE) scale after a treadmill walk of 5 min: range 6−20. A higher score indicates more exertion and higher perceived fatigability.	Chronic Inflammation	Fasting serum samples; enzyme-linked immunosorbent essay for interleukine-6 concentrations	Increase in log IL-6 corresponds to perceived exertion increase in the continuous analysis; in the categorical analysis higher IL-6 levels reported higher perceived exertion at the baseline and over the time
Hoekstra C. et al., 2023	Self-Perceived Fatigue (SPF): score 0−10 at the question "How tired do you feel at this moment?"	Chronic Inflammation	Blood sample for inflammatory markers were taken as part of routine clinical care upon admission and CPR measurement were extracted from reports	Participants with abnormal CRP values had higher self-perceived fatigue score, but not statistically significant
Hughes A. et al.; 2017	Short Form Health Survey (SF-12): fatigue is indexed by a single item: ‘How much of the time during the past 4 weeks did you have a lot of energy?’	Chronic Inflammation	Blood sample taken during the interview; CRP was analysed from serum using the high-sensitivity N Latex CRP mono Immunoassay	Medium and high CRP were associated with fatigue in participants aged 61–98, only high CRP was associated with fatigue in participants aged 44–60, and neither was associated with fatigue in participants aged 16–43. Longitudinally, both medium and high CRPs predicted elevated fatigue, and high CRP predicted odds of new-onset fatigue in participants aged 61–98
White J. et al., 2016	1 question from the Centre for Epidemiologic Studies-Depression scale (CES-D): the participants were asked to consider their experience in the past week related to two statements: (a) “I feel that everything I did was an effort ". Possible answers were (a) rarely or none of the time (less than 1 day), (b) some or a little of the time (1–2 days), (c) occasionally or a moderate amount of time (3–4 days), (d) all of the time (5–7 days). Those reporting three or more days were classified as being fatigued	Chronic Inflammation	Blood sample; CRP was measured using the N Latex CRP mono Immunoassay	In older adults that are not taking antidepressant drugs there is an association between circulating CRP levels and fatigue
Avlund K. et al., 2012	Short Form Health Survey (SF-12): fatigue is indexed by a single item: ‘How much of the time during the past 4 weeks did you have a lot of energy?’	Chronic Inflammation	Blood sample	Total number of lymphocytes was associated with fatigue in old sample; the association was attenuated when adjusting for physical activity and disability
Vestergaard S. et al., 2009	2 questions from the Centre for Epidemiologic Studies-Depression scale (CES-D): the participants were asked to consider their experience in the past week related to two statements: (a) “I feel that everything I did was an effort” and (b) “I could not get going.” Possible answers were (a) rarely or none of the time (less than 1 day), (b) some or a little of the time (1–2 days), (c) occasionally or a moderate amount of time (3–4 days), (d) all of the time (5–7 days). Those reporting three or more days to either question were classified as being fatigued.	Chronic Inflammation	Blood sample after 12 h fasting; High-sensitivity CRP was measured using the BNII nephelometer; Serum IL-6 was assessed with ultrasensitive enzyme-linked immunosorbent assay; Serum TNF- α was measured by an ultrasensitive solid-phase sandwich ELISA	Fatigued men and women had higher CRP levels; no association with other biomarkers
Valentine R. J. et al., 2009	Cohen-Hoberman Inventory of Physical Symptom (CHIPS) questionnaire: 2 items assess if the individuals have experienced "constant fatigue" and "feeling low energy" over the last é weeks rating on 5-point scale, higher scores reflect more fatigue	Chronic Inflammation	Blood sample after overnight fasting; CRP was measured using a high sensitivity ELISA kits; body composition was measured using dual energy X-ray absorptiometry (DXA)	Adiposity was positively associated with constant fatigue in women; CRP was related to fatigue in women; CRP and adiposity were significant greater in fatigued women than in non-fatigued; inflammation was a significant independent predictor factor of fatigue in women
Zang Q. et al., 2022	Fatigue Severity Scale (FSS): 9-items measuring fatigue through its effect on daily functioning, higher scores indicate greater fatigue	Dysbiosis	Faecal samples collected within 2 days after admission; sequencing was performed with Illumina	Bacteroides and Flavonifractor species were positively correlated with fatigue in both patients and controls

Legend: TNF-α = Tumour Necrosis Factor α, LTL = leukocyte telomer length, OCR = oxygen consumption rate, ATP = adenosine tri-phosphate, AMPK = 5’ adenosine monophosphate-activated protein kinase, CRP = C-reactive protein, IL-6 = interleukine-6, Hsp70 = Heat Shock Protein 70, BMI = body mass index, WBC = white blood cells.

#### 3.1.1 Primary drivers of biological ageing

Primary drivers are biological processes leading to cellular dysfunction during ageing, including genetic and epigenetic alterations as well as the regulation of protein synthesis [11]. We found two studies that explored the relationship between self-perceived fatigue and primary hallmarks of ageing, specifically focusing on genomic instability [16] and telomere attrition [17], respectively.

Regarding genomic instability, a study involving 253 participants - including both oncology outpatients and their healthy caregivers - showed that a genetic variation in a promoter polymorphism in the Tumour Necrosis Factor alpha (TNFA-308G>A [rs1800629]) was associated with overall lower fatigue ratings cross-sectionally and longitudinally, not only among cancer-patients but also in their caregivers [16].

Regarding telomere length, evidence from the Long-Life Family Study in Chicago, an ongoing population study enrolling community-dwelling older adults, showed that shorter leukocyte telomere length (LTL) was correlated with higher self-reported fatigue in older adults. In a longitudinal analysis, shorter LTL was associated with higher scores for fatigue over an 8-year follow-up [17]. Telomere shortening leads to cycle arrest and apoptosis (12), with a consequent reduction in myogenic progenitor and functional tissue (20). Telomere attrition may also contribute to low-grade inflammation and oxidative stress (21).

We did not find any studies addressing epigenetic alterations, loss of proteostasis and disabled macroautophagy in older adults. Although several studies [[18], [19], [20]] have reported a relation between Epigenetic Age Acceleration (EAA) and fatigue severity in cancer population, no studies investigated this association in older individuals without oncologic comorbidities. At the same time, loss of proteostasis has been linked to fatigue in the context of neurodegenerative disease, but these results cannot be generalised to the broader older population [21,22]. Given the critical role of these pathways, particularly in identifying potential therapeutic targets, further investigations are warranted to elucidate the molecular alterations that occur in older adults in the absence of major comorbidities.

In summary, the very limited evidence investigating the relationship between primary drivers of biological ageing and fatigue suggests that both genomic instability and LTL are associated to self-perceived fatigue.

#### 3.1.2 Markers of antagonistic response

The antagonistic response category of the hallmarks of ageing comprises compensatory pathways that are activated in response to primary cellular damage. Initially, this compensation acts as a protective mechanism, but the dysregulation of these mechanisms beyond a certain threshold plays a crucial role in determining the intensity or duration of negative stimuli [11]. Deregulated nutrient sensing, mitochondrial dysfunction and cellular senescence are the hallmarks of an antagonistic response.

Nutrient sensing, the cellular process that enables adaptation to specific nutrient cues, such as glucose, amino acids, and lipids, is among the most conserved regulatory mechanisms in evolution [11]. Although the biological mechanisms linking deregulated nutrient sensing to fatigue are not fully understood, results from small cross-sectional studies suggest a potential role for the regulation of energetic expenditure [23] and overnutrition [11]. In an obese population that also included patients over 65 years old, fatigue correlated with body mass excess and glucose metabolism impairment, especially in women [24]. Moreover, higher serum levels of leptin, a key hormone in the regulation of energetic expenditure [23], were associated with greater fatigue severity independently of body weight among community-dwelling older adults with cardiovascular risk factors [25] and healthy women [26]. These gindigs are consistent with preclinical evidence showing that leptin administration in mice inhibits serotoninergic activity and leads to increased fatigue and reduced capacity for sustained physical performance [27,28].

Mitochondrial activity may be related to fatigue through impairment of the electron transport chain and early reliance on anaerobic metabolism, consistent with the essential role of mitochondria in maintaining cellular homeostasis and generating energy [10]. Age-related fatigue has been associated with reduced mitochondrial oxidative capacity and earlier use of anaerobic metabolism ina longitudinal study of 544 community-dwelling older adults without major comorbidities [29]. Additional cross-sectional evidence showed a significant reduction of cytochrome C oxidase, AMPK (5’ adenosine monophosphate-activated protein kinase) activation, lower levels of mitochondrially-localised Sirt3 protein levels, along with decreased electron transport chain efficiency, in sedentary older adults V [30]. Similarly, lower oxygen consumption rate (OCR) and lower adenosine triphosphate (ATP) turnover were observed in hospitalised geriatric patients with age-related fatigue [31]. This supports the hypothesis that mitochondrial processes may represent potential therapeutic targets for interventions aimed at reducing fatigue. In line with this hypothesis, a recent randomised clinical trial showed that elamipretide - a cardiolipin-binding compound targeting the inner mitochondrial membrane to improve oxidative phosphorylation [11] - increased skeletal muscle ATP production in healthy older adults. However, elamipretide did not improve muscle endurance, indicating that the clinical implications of targeting mitochondrial dysfunction remain incompletely understood [32].

No studies have specifically addressed the relationship between cellular senescence and self-perceived fatigue.

In summary, our findings indicate a link between antagonistic response and self-perceived fatigue, with mitochondrial dysfunction showing the strongest association, followed by deregulated nutrient sensing.

#### 3.1.3 Integrative markers

As the final category, integrative markers emerge when the damage caused by the primary and antagonistic markers passed a certain threshold and cannot be compensated. This category includes: stem cell exhaustion, altered intercellular communication, chronic inflammation and dysbiosis [11].

We identified numerous studies examining the role of inflammatory markers - such as interleukin-6 (IL-6) or C-reactive protein (CRP) - in the onset and severity of fatigue.

Longitudinal evidence supports a link between IL-6 and fatigue. In particular higher circulating IL-6 levels were associated with greater fatigue severity in a cohort of 985 middle-to-late-life adults [33], and IL-6 levels at 60–64 years predicted greater fatigue after four years of follow-up in 1,580 community-dwelling individuals [34]. Similarly, in two small cross-sectional studies, higher IL-6 levels were associated with greater self-perceived fatigue among 77 nursing home residents [35] and 40 older adults without systemic chronic inflammation, defined by low serum CRP levels [36]. In addition, in 55 community-dwelling older adults exposed to cognitive stimulation, fatigue was associated with higher baseline IL-6 levels and greater IL-6 increase after stimulation [37]. Longitudinal evidence on CRP points in a similar direction: elevated serum CRP levels predicted new-onset fatigue in an age-dependent manner in a longitudinal study with 10,606 community-dwelling adults [38]. Cross-sectionally, higher CRP levels have been associated with greater fatigue in healthy community-dwelling adults [39] and hospitalised older adults [40] older adults. Consistently, in a cohort of 5,909 community-dwelling older adults, higher CRP levels were associated with somatic symptoms of depression - including fatigue - among participants not taking antidepressant [41]. Only a single small cross-sectional study including 135 sedentary women reported no association between fatigue and inflammatory markers, specifically IL-6 or TNFα receptor 1 [42].

Both IL-6 and CRP interact with adiposity and overnutrition in the context of fatigue. In particular, inflammatory markers appeared to play a moderating role both in 167 individuals undergoing weight loss [43] and in 182 community-dwelling older adults in relation to physical performance impairment [44]. Furthermore, increased inflammation may lead to decline in immune function with ageing, with consequent modification in blood cell count [11]; changes in blood cell counts may be associated to fatigue. Indeed, reductions in total leukocytes, lymphocytes and neutrophils counts were associated with fatigue in 314 community-dwelling older adults, when compared to a younger control group [45].

Overall, inflammatory cytokines—and chronic inflammation more broadly— may contribute to the onset of fatigue through the induction of sickness behavior, either after crossing the blood–brain barrier or via the activation of specific immune-to-brain communication pathways [46,47]. In this context, CRP has been investigated in several studies on specific clinical conditions, which reported a longitudinal CRP-fatigue association also in middle-aged populations [[48], [49], [50]]. However, in community-dwelling individuals, the role of these markers remains unclear, as current evidence is largely based on small and cross-sectional studies. Further research is therefore needed to clarify their contribution, including their potential interaction with obesity [[51], [52], [53], [54]].

For what concerns altered intercellular communication, we found a cross-sectional study among 284 community-dwelling older adults showing that impairment in the metabolism of monoamine after immune stimuli, such as increased tryptophan catabolism and altered phenylalanine turnover, was related with increased fatigue [47].

Regarding dysbiosis, a cross-sectional study suggested that higher prevalence of *Bacteroides* and *Flavonifractor* species was positively correlated to fatigue severity in both patients with depressive disorder and healthy controls [55]. *Flavonifractor* is a gram-positive anaerobic butyrate-producing bacterium and it is associated to various diseases including cancer, inflammatory bowel disease and bipolar disorder [56,57]. Nevertheless, butyrate could downregulate proinflammatory cytokines and produce an anti-inflammatory effect [58]. *Bacteroides* species are gram-negative anaerobic bacteria associated with oxidative stress response via scavenging enzymes [59]. The dual role of *Flavonifractor* species and the seemingly contradictory effects of *Bacteroides* species warrant further clarification.

In summary, integrative markers and, in particular chronic inflammation, constitute one of the most extensively explored hallmarks of ageing in relation to fatigue. The roles of the gut-microbiota-brain axis and the intercellular communication remain insufficiently understood and requires further investigation.

### 3.2 Muscle fatigability and hallmarks of ageing

Muscle fatigability is a relatively new concept in geriatric research, with no gold-standard measures or universally accepted definitions in either research or clinical practice [15,60]. It is generally agreed that muscle fatigability should capture changes in self-perceived fatigue during physical activity and that tasks should be standardised for comparability [60]. A summary of studies investigating muscle fatigability and hallmarks of ageing is provided in Table 3. Research directly examining the link between hallmarks of ageing and muscle fatigability remains scarce.

**Table 3:** Summary of findings from studies on muscle fatigability.

First author, year	Fatigue assessment	Hallmark of Aging	Hallmark of Aging assessment	Investigation results
Yuichiro O. et al., 2024	Total power: total autonomic nerve activity obtained by measuring electrocardiogram; low values indicate more fatigability	Cellular Senescence	Erythrocyte counts, senescent erythrocyte rate, damaged erythrocyte rate, erythrocyte sedimentation rate	Accumulation of senescent erythrocytes in the elderly contributes to the onset of fatigability
Soendenbroe C. et al., 2022	Maximal Voluntary Contraction (MCV) in a dynamometer	Stem Cell Exhaustion	Biopsies from the middle portion of vastus lateralis muscle from both legs; Myofiber cross-sectional area, type area and composition percentage on composite images; Satellite cells manually quantified on composite images	Fatigue resistance and higher number of type II fibre-associated satellite cells in skeletal muscle in elderly population is associated to life-long recreational exercise
Alway S. E. et al., 2017	Fatigue Index (FI): the decline in torque over 32 isokinetic contractions, lower scores correspond to high muscle fatigue	Stem Cell Exhaustion	Needle biopsies of the vastus lateralis muscle, the relative number of mtDNA copies was quantified by real time PCR, Satellite cells were identified by specific antibodies	Subjects who exercise and consume resveratrol improve their fatigue resistance and have more nuclei of satellite cells compared to control. In this group there was an increase of the number of nuclei of satellite cells
Bautmans I. et al., 2006	Fatigue resistance: time (in seconds) during which grip strength dropped to 50% of its maximum. The subject was asked to squeeze the large bulb of the vigorimeter as hard as possible: the shoulder was adducted and neutrally rotated, elbow flexed at 90 °, forearm in neutral position and wrist in slight extension (0 to 30 °)	Chronic Inflammation	Blood sample for CRP and IL-6	Muscle Fatigue resistance in male participants with no inflammatory activity correlates positively with circulating levels of IL-6 levels
Bautmans I. et al., 2005	Fatigue resistance: time (in seconds) during which grip strength dropped to 50% of its maximum. The subject was asked to squeeze the large bulb of the vigorimeter as hard as possible: the shoulder was adducted and neutrally rotated, elbow flexed at 90 °, forearm in neutral position and wrist in slight extension (0 to 30 °); higher scores correspond to get tired less easily	Chronic Inflammation	Blood sample for CRP, fibrinogen and IL-6	Reduced muscle fatigue resistance in hospitalized geriatric patients with inflammation was significantly related to the concentration of CRP protein and IL-6
Bautmans I. et al., 2008	Fatigue resistance: time (in seconds) during which grip strength dropped to 50% of its maximum. The subject was asked to squeeze the large bulb of the vigorimeter as hard as possible: the shoulder was adducted and neutrally rotated, elbow flexed at 90 °, forearm in neutral position and wrist in slight extension (0 to 30 °); higher scores correspond to get tired less easily	Chronic Inflammation	Blood sample for IL-6, TNF-α and Hsp70	Higher levels of circulating TNF-α were related to worse fatigue resistance in the male residents; Residents with both high IL-6 and Hsp70 serum levels had significantly poorer fatigue resistance
Silva J. P. et al., 2010	Muscular fatigue index (MFI): the percentage of work declined during 21 maximal repetition of knee extension in an angular velocity of 180 °/s and a range of motion of 90 °; higher scores indicate a higher level of muscular fatigue	Chronic Inflammation	Blood sample for plasma concentrations of the inflammatory mediators IL-6 and soluble receptor of TNF-α	No association between self-muscle fatigue and IL-6 levels
Sorensen J. R., et al., 2018	Rate of functional decline: average knee extensor power output per set through the duration of the exercise protocol	Chronic Inflammation	Muscle biopsies were taken at the baseline and then 3, 24 and 72 h post-exercise from the non-exercised leg	Older group demonstrated significantly greater fatigue resistance through the exercise session than the young subjects; Aging appears to potentiate the premature activation of TGF-β signalling pathway mediating collagen expression; no difference in intramuscular inflammation at the baseline; the acute response to damage was marked by different timing in MCP-1 expression
Arnold P. et al., 2017	Fatigue resistance: number of repetitions till the strength dropped to 50% of its maximum. The subject was asked to adducte the thumb of the dominant side after the forearm and the hand were placed in supination in a thermoplastic open cast with other fingers and wrist velcro taped to the cast	Chronic Inflammation	Serum sample was collected before fatigue assessment; 25 different cyto/chemokines were simultaneously measured by multiplex bead immunoassay	Higher levels of inflammatory markers were related to worse muscle contractility

Legend: TNF-α = Tumour Necrosis Factor α, CRP = C-reactive protein, IL-6 = interleukine-6, Hsp70 = Heat Shock Protein 70, BMI = body mass index, WBC = white blood cells, mtDNA = mithocondrial DNA, TGF-β = transforming growth factor beta.

Cellular senescence, a marker of antagonistic response, is a state of permanent cell-cycle arrest triggered by various forms of damage [61]. This process leads to the release of senescence-associated secretory phenotype (SASP), which affects both the microenvironment - by activating immune cells through cytokine and chemokine secretion and inducing paracrine senescence in neighbouring cells [62] – and the general environment, by entering the bloodstream [63]. A small cross-sectional study among 22 healthy old males suggested that the accumulation of senescent erythrocytes due to the suppression Hbbb1 mitochondrial-RNA expression by SASP factors in bone marrow (e.g., interleukin-1α), was associated to muscle fatigability [64].

Stem cell exhaustion is one of the integrative hallmarks of ageing [11]. With advancing age, tissue renewal decreases and also the process of tissue repair after injury becomes impaired [65]. Evidence linking stem cell function to fatigability is limited, with two small studies. In the first, 16 older adults with a history of lifelong physical activity were compared with 15 sedentary peers: the active group exhibited a higher number of satellite cells associated with type II fibres and lower fatigability, despite no significant difference in satellite cell differentiation capacity [66]. Furthermore, a clinical trial involving 15 older adults demonstrated that resveratrol supplementation - a plant-derived polyphenol with anti-inflammatory and anti-fibrotic properties [67] - enhanced resistance to muscle fatigue and increased satellite cell numbers relative to placebo [68]. These findings, which simultaneously suggest an increase in satellite cell nuclei but no consistent change in differentiation potential, underscore the preliminary and partially conflicting nature of current evidence and highlight the need for further research.

Several studies have investigated the role of chronic inflammation in muscle fatigability, with the most substantial evidence coming from two observational longitudinal studies. Both studies highlight a role for monocyte chemoattractant protein-1 (MCP-1) in age-related muscle fatigability. In 10 hospitalised geriatric patients and 9 community-dwelling controls, impaired muscle contractility induced by a fatigue protocol was associated with significantly elevated circulating MCP-1, with a non-significant trend for Interleukin 1 (IL-1) receptor antagonist [69]. Similarly, in 19 older community-dwelling adults, exercise-induced muscle damage triggered an exaggerated inflammatory response, characterised by higher MCP-1 expression alongside increased NFkB (Nuclear Factor Kappa-light-chain-enhancer of Activated B Cells, p65) and a trend toward elevated interleukin-8, despite similar baseline intramuscular inflammation [70]. These findings suggest that MCP-1 may mediate the inflammatory contribution to muscle fatigability in ageing muscle. In contrast to self-perceived fatigue, the association between IL-6 and muscle fatigability is largely inconsistent and based only on small cross-sectional studies. Available evidence regarding IL-6 is therefore limited and inconsistent. Two small studies found associations between IL-6 and muscle fatigability – one including 63 hospitalised patients [71], in which CRP also played a role, and another with 40 community-dwelling older adults [36]. In contrast, no association was observed among 77 nursing residents without basal inflammation based on CRP blood levels [35] or among 135 older women [42].

Overall, the evidence on muscle fatigability is considerably more limited than that on self-perceived fatigue and mainly concerns cellular senescence, stem cell exhaustion, and chronic inflammation. Among these, chronic inflammation represents the most extensively investigated hallmark of ageing in this context, although further clarification is needed. Based on limited evidence available, IL-1α inhibition would alleviate fatigue in older adults [72]. Further research on the IL-1α-fatigue relationship in older people is therefore warranted to address an important biogerontology knowledge gap [64].

## 4 Final considerations

**Fig. 1:**
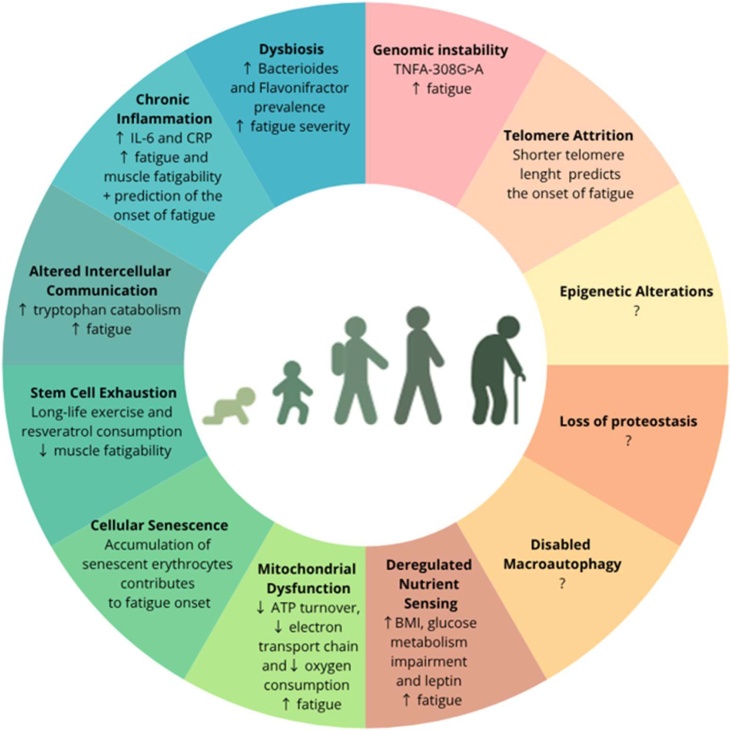
Association between the 12 hallmarks of aging and Fatigue.

This narrative review provides evidence of a relationship between several hallmarks of ageing and fatigue, defined as both self-reported fatigue and muscle fatigability (Fig. 1). Among them, mitochondrial dysfunction and chronic inflammation appear to be the most extensively studied biological drivers of ageing, providing some evidence that dysregulation in these hallmarks is associated with increased fatigue. Existing evidence, although limited, suggest that some markers of genomic instability, telomere attrition, deregulated nutrient sensing, and stem cell exhaustion, were related to fatigue; evidence for each of these hallmarks was supported by at least one longitudinal study. In contrast, cellular senescence, altered intercellular communication, and dysbiosis were only examined in cross-sectional studies. Moreover, no studies were identified that examined epigenetic alterations, proteostasis dysfunction, or impaired macroautophagy in relation to fatigue in older adults without specific comorbidities.

At the cellular level, fatigue may share similar mechanistic pathways with appetite loss, a frequent age-related symptom, which is considered a key element for promoting functioning and preventing dependency. Studies have shown that loss of appetite and fatigue are clinical symptoms that may appear together, suggesting common biological pathways [73]. In this context, the Growth Differentiation Factor (GDF-15), which plays a significant role in cellular stress and energy homeostasis, appears as a potential candidate biomarker linking appetite loss and fatigue in older adults. Indeed, GDF-15 is associated with cachexia in cancer, chemotherapy-induced anorexia and mitochondrial disorders [74], conditions that are often characterised by fatigue. GDF-15-related mechanisms have a role in linking appetite loss and ageing through mitochondrial dysfunction and leptin alterations [75]. In patients with mitochondrial disease, GDF-15 was also associated with fatigue severity [76]. Therefore, the potential links between appetite loss and fatigue deserves further investigation. This is particularly important since it may lead to the inclusion of fatigue assessment/treatment in routine clinical care of older people. Indeed, appetite loss is one of the core constructs assessed in the Integrated Care for Older People (ICOPE), a function-centered healthcare pathway developed by the World Health Organization [77]. In ICOPE, intrinsic capacity (all the mental and physical capacities of an individual) is the central element that triggers the different steps of the ICOPE pathway; it is composed of six domains that play crucial roles in the preservation of independent living: locomotion, cognition, psychology, vitality (including appetite loss), hearing and vision [78]. In recent literature, measures of fatigue and muscle fatigability have been used as proxies for evaluating the vitality domain of intrinsic capacity, which is linked to body energy and metabolism [8]. From this framework, fatigue may be considered a key component of vitality, even though it may also manifest across other intrinsic capacity domains. Notably, individuals reporting higher levels of mental fatigue tend to exhibit a greater burden of depressive symptoms, while those with more pronounced physical fatigue often show impairments in the locomotion domain [79,80]. Using fatigue as a key element of the domain of vitality will require standardisation/improvement of fatigue assessment tools [8]. The development of a fatigue index, integrating self-perceived fatigue and muscle fatigability and accounting also for physical disability, appears to be urgently needed [8].

The evidence gathered in this review suggests fatigue may be modulated by biological drivers of aging, in particular, mitochondrial function and chronic inflammation. However, the findings of the present review must be carefully interpreted due to the several methodological limitations of the original investigations included. First, most of the included studies had small sample sizes. Second, many studies are cross-sectional or have short followup durations, thereby limiting the generalisability of the findings. Third, a huge heterogeneity in fatigue definitions, assessment protocols and study populations were found, reducing comparability. A key limitation may be attributed to a lack of gold standard for measuring self-reported fatigue and muscle fatigability, which complicates direct comparisons across studies [8].

Several research and clinical perspective/directions can be drawn from this work. First, considering the intricate interconnection among hallmarks of ageing, future research on fatigue should address multiple hallmarks simultaneously. For example, mitochondrial dysfunction rarely occurs in isolation but is often accompanied by increased reactive oxygen species, chronic inflammation, disrupted nutrient sensing, and impaired autophagy [81]. A more integrative approach may therefore yield richer mechanistic insight, in particular when using a longitudinal study design that allow to establish temporal associations (e.g., hallmarks of ageing associated with incident fatigue or the evolution of fatigue severity). From a Geroscience perspective, future studies should explore the relationship between fatigue and biological ageing processes within the broader framework of intrinsic capacity, particularly within the energy domain and biological ageing [82]. In this context, the identification of biological markers linked to ageing hallmarks may contribute to improving fatigue assessment in ageing research and to developing more objective measures alongside self-reported symptoms. A key observation is that pathological conditions can generate fatigue through distinct mechanisms. For example, inflammatory pathways contribute to fatigue during influenza or COVID-19 infections, whereas fatigue associated with anaemia is primarily driven by impaired energy metabolism. Conversely, lifestyle factors such as physical activity—known to influence the biology of ageing—can help mitigate fatigue. Future investigations should examine whether interventions targeting the cellular and molecular pathways of ageing can prevent or mitigate fatigue severity. Some emerging therapeutic strategies already support this approach. For instance, a pharmacological agent against GDF-15 activity – Ponsegromab, a humanised monoclonal antibody - has been shown to improve cancer-related cachexia and its associated features, including both fatigue and appetite loss [83]. Similarly, compounds such as resveratrol [67] and elamipretide [32], although still under investigation, may represent promising candidates for targeting ageing-related mechanisms underlying fatigue. Advancing this line of research may ultimately help to bridge the existing knowledge gap between ageing-related biological mechanism and therapeutic strategies for fatigue, which currently remain limited and largely based on behavioural interventions (i.e., mindfulness, muscle relaxation, yoga) [84].

## Authorship contribution statement

Anna Ronca: Conceptualization, Methodology, Investigation, Writing- Original draft preparation; Federico Bellelli: Supervision, Reviewing and Editing; Marco Proietti: Reviewing and Editing; Yves Rolland: Reviewing and Editing; Bruno Vellas: Conceptualization, Reviewing and Editing; Philipe De Souto Barreto: Conceptualization, Methodology, Supervision, Reviewing and Editing.

## Ethical statement

As this study is a review, approval from institutional ethics committee was not required.

## Declaration of Generative AI and AI-assisted technologies in the writing process

Generative artificial intelligence was used exclusively for language editing and proofreading. No scientific content was generated.

All other authors declare they have no conflict of interest

## Data statement

Not applicable

## Declaration of competing interest

MP reports small speaker’s fees from Pfizer and BMS/JJ Alliance, consulting activities for Regeneron Pharmaceuticals, and role of Italian national Principal Investigator of the AFFIRMO project on multimorbidity in atrial fibrillation, which has received funding from the European Union’s Horizon 2020 research and innovation program under grant agreement No 899871.

## References

[1] Yu D.S.F., Lee D.T.F., Man N.W. (2010). Fatigue among older people: a review of the research literature. Int J Nurs Stud.

[2] Morelli V. (2011). Fatigue and chronic fatigue in the elderly: definitions, diagnoses, and treatments. Clin Geriatr Med.

[3] Knoop V., Cloots B., Costenoble A., Debain A., Vella Azzopardi R., Vermeiren S. (2021). Fatigue and the prediction of negative health outcomes: a systematic review with meta-analysis. Ageing Res Rev.

[4] Lopez-Leon S., Wegman-Ostrosky T., Perelman C., Sepulveda R., Rebolledo P.A., Cuapio A. (2021). More than 50 long-term effects of COVID-19: a systematic review and meta-analysis. Sci Rep.

[5] Hu T., Wang F., Duan Q., Zhao X., Yang F. (2025). Prevalence of fatigue and perceived fatigability in older adults: a systematic review and meta-analysis. Sci Rep.

[6] Gems D., Partridge L. (2013). Genetics of longevity in model organisms: debates and paradigm shifts. Annu Rev Physiol.

[7] Zhou H., Yu W., Lei J., Chang R., Cheng Y., Wang G. (2026). Pathophysiological mechanisms of fatigue and multidisciplinary management strategies (Review). Exp Ther Med.

[8] Knoop V., Mathot E., Louter F., Beckwee D., Mikton C., Diaz T. (2023). Measurement properties of instruments to measure the fatigue domain of vitality capacity in community-dwelling older people: an umbrella review of systematic reviews and meta-analysis. Age Ageing.

[9] Moreh E., Jacobs J.M., Stessman J. (2010). Fatigue, function, and mortality in older adults. J Gerontol A Biol Sci Med Sci.

[10] Angioni D., Raffin J., Ousset P.-J., Delrieu J., de Souto Barreto P. (2023). Fatigue in Alzheimer’s disease: biological basis and clinical management-a narrative review. Aging Clin Exp Res.

[11] López-Otín C., Blasco M.A., Partridge L., Serrano M., Kroemer G. (2023). Hallmarks of aging: an expanding universe. Cell.

[12] Carmichael C., Gousset C., Burns D., Miller J., Van Tomme S., Kitchen H. (2025). Fatigue experience in oncology; a targeted qualitative literature review and novel patient-centric conceptual model. Adv Ther.

[13] Diaz-Quiroz M., Chicue-Cuervo P.C., Garcia-Moreno L., Gaviria-Carrillo M., Talero-Gutierrez C., Palacios-Espinosa X. (2025). Fatigue in multiple sclerosis: a scoping review of pharmacological and nonpharmacological interventions. Mult Scler J Exp Transl Clin.

[14] Institute of Medicine (2015).

[15] Glynn N.W., Santanasto A.J., Simonsick E.M., Boudreau R.M., Beach S.R., Schulz R. (2015). The Pittsburgh fatigability scale for older adults: development and validation. J Am Geriatr Soc.

[16] Aouizerat B.E., Dodd M., Lee K., West C., Paul S.M., Cooper B.A. (2009). Preliminary evidence of a genetic association between tumor necrosis factor alpha and the severity of sleep disturbance and morning fatigue. Biol Res Nurs.

[17] Katz R., Gay E.L., Kuipers A.L., Lee J.H., Honig L.S., Christensen K. (2022). Association of leukocyte telomere length with perceived physical fatigability. Exp Gerontol.

[18] Le C., Lewis M., Harris C.S., Berger L., Chavez-Iglesias E., Morse L. (2025). A data-driven epigenetic characterization of morning fatigue severity in oncology patients receiving chemotherapy: associations with epigenetic age acceleration, blood cell types, and expression-associated methylation. Cancer Med.

[19] Xiao C., Beitler J.J., Peng G., Levine M.E., Conneely K.N., Zhao H. (2021). Epigenetic age acceleration, fatigue, and inflammation in patients undergoing radiation therapy for head and neck cancer: a longitudinal study. Cancer.

[20] Yang G.S., Yang K., Weaver M.T., Lynch Kelly D., Dorsey S.G., Jackson-Cook C.K. (2022). Exploring the relationship between DNA methylation age measures and psychoneurological symptoms in women with early-stage breast cancer. Support Care Cancer.

[21] Gindri I. de M., Ferrari G., Pinto L.P.S., Bicca J., Dos Santos I.K., Dallacosta D. (2024). Evaluation of safety and effectiveness of NAD in different clinical conditions: a systematic review. Am J Physiol Endocrinol Metab.

[22] Van Doeselaer L., Colman K., Crosiers D., Dijkstra F. (2025). Effect of patient education interventions on non-motor symptoms and disease outcomes in Parkinson’s disease: a systematic review. Acta Neurol Belg.

[23] Breit S.N., Manandhar R., Zhang H.-P., Lee-Ng M., Brown D.A., Tsai V.W.-W. (2023). GDF15 enhances body weight and adiposity reduction in obese mice by leveraging the leptin pathway. Cell Metab.

[24] Lafortuna C.L., Prinelli F., Adorni F., Agosti F., De Col A., Sartorio A. (2013). Effect of mechanical and metabolic factors on motor function and fatigue in obese men and women: a cross-sectional study. J Endocrinol Invest.

[25] Kurajoh M., Kadoya M., Morimoto A., Naka M., Miyoshi A., Kanzaki A. (2016). Plasma leptin concentration is associated with fatigue severity in patients with cardiovascular risk factors - HSCAA study. Psychoneuroendocrinology.

[26] Stringer E.A., Baker K.S., Carroll I.R., Montoya J.G., Chu L., Maecker H.T. (2013). Daily cytokine fluctuations, driven by leptin, are associated with fatigue severity in chronic fatigue syndrome: evidence of inflammatory pathology. J Transl Med.

[27] Calapai G., Corica F., Corsonello A., Sautebin L., Di Rosa M., Campo G.M. (1999). Leptin increases serotonin turnover by inhibition of brain nitric oxide synthesis. J Clin Invest.

[28] Wilson W., Maughan R. (1992). Evidence for a possible role of 5-hydroxytryptamine in the genesis of fatigue in man: administration of paroxetine, a 5-HT re-uptake inhibitor, reduces the capacity to perform prolonged exercise. Exp Physiol.

[29] Liu F., Wanigatunga A.A., Zampino M., Knuth N.D., Simonsick E.M., Schrack J.A. (2021). Association of mitochondrial function, substrate utilization, and anaerobic metabolism with age-related perceived fatigability. J Gerontol A Biol Sci Med Sci.

[30] Wawrzyniak N.R., Joseph A.M., Levin D.G., Gundermann D.M., Leeuwenburgh C., Sandesara B. (2016). Idiopathic chronic fatigue in older adults is linked to impaired mitochondrial content and biogenesis signaling in skeletal muscle. Oncotarget.

[31] Herpich C., Franz K., Klaus S., Müller-Werdan U., Ost M., Norman K. (2021). Age-related fatigue is associated with reduced mitochondrial function in peripheral blood mononuclear cells. Exp Gerontol.

[32] Roshanravan B., Liu S.Z., Ali A.S., Shankland E.G., Goss C., Amory J.K. (2021). In vivo mitochondrial ATP production is improved in older adult skeletal muscle after a single dose of elamipretide in a randomized trial. PLoS One.

[33] Wanigatunga A.A., Varadhan R., Simonsick E.M., Carlson O.D., Studenski S., Ferrucci L. (2019). Longitudinal relationship between interleukin-6 and Perceived fatigability among well-functioning adults in mid-to-late life. J Gerontol A Biol Sci Med Sci.

[34] Cooper R., Popham M., Santanasto A.J., Hardy R., Glynn N.W., Kuh D. (2019). Are BMI and inflammatory markers independently associated with physical fatigability in old age?. Int J Obes (Lond).

[35] Bautmans I., Njemini R., Predom H., Lemper J.-C., Mets T. (2008). Muscle endurance in elderly nursing home residents is related to fatigue perception, mobility, and circulating tumor necrosis factor-alpha, interleukin-6, and heat shock protein 70. J Am Geriatr Soc.

[36] Bautmans I., Gorus E., Njemini R., Mets T. (2007). Handgrip performance in relation to self-perceived fatigue, physical functioning and circulating IL-6 in elderly persons without inflammation. BMC Geriatr.

[37] Lin F., Roiland R., Polesskaya O., Chapman B., Johnson M., Brasch J. (2014). Fatigability disrupts cognitive processes’ regulation of inflammatory reactivity in old age. Am J Geriatr Psychiatry.

[38] Hughes A., Kumari M. (2018). Age modification of the relationship between C-reactive protein and fatigue: findings from Understanding Society (UKHLS). Psychol Med.

[39] Valentine R.J., McAuley E., Vieira V.J., Baynard T., Hu L., Evans E.M. (2009). Sex differences in the relationship between obesity, C-reactive protein, physical activity, depression, sleep quality and fatigue in older adults. Brain Behav Immun.

[40] Hoekstra C., Swart M., Bautmans I., Melis R., Peeters G. (2023). Association between muscle fatigability, self-perceived fatigue and C-reactive protein at admission in hospitalized geriatric patients. Int J Environ Res Public Health.

[41] White J., Kivimäki M., Jokela M., Batty G.D. (2017). Association of inflammation with specific symptoms of depression in a general population of older people: The English Longitudinal Study of Ageing. Brain Behav Immun.

[42] Silva J.P., Pereira D.S., Coelho F.M., Lustosa L.P., Dias J.M.D., Pereira L.S.M. (2011). Clinical, functional and inflammatory factors associated with muscle fatigue and self-perceived fatigue in elderly community-dwelling women. Rev Bras Fisioter.

[43] Nicklas B.J., Beavers D.P., Mihalko S.L., Miller G.D., Loeser R.F., Messier S.P. (2016). Relationship of objectively-measured habitual physical activity to chronic inflammation and fatigue in middle-aged and older adults. J Gerontol A Biol Sci Med Sci.

[44] Valentine R.J., Woods J.A., McAuley E., Dantzer R., Evans E.M. (2011). The associations of adiposity, physical activity and inflammation with fatigue in older adults. Brain Behav Immun.

[45] Avlund K., Hokland M., Mehlsen M.Y., Thomsen D.K., Viidik A., Ekmann A. (2012). Differential associations between white blood cell counts and fatigue in young and older adults. Aging Clin Exp Res.

[46] Dantzer R., Kelley K.W. (2007). Twenty years of research on cytokine-induced sickness behavior. Brain Behav Immun.

[47] Capuron L., Schroecksnadel S., Féart C., Aubert A., Higueret D., Barberger-Gateau P. (2011). Chronic low-grade inflammation in elderly persons is associated with altered tryptophan and tyrosine metabolism: role in neuropsychiatric symptoms. Biol Psychiatry.

[48] Cho H.J., Seeman T.E., Bower J.E., Kiefe C.I., Irwin M.R. (2009). Prospective association between C-reactive protein and fatigue in the coronary artery risk development in young adults study. Biol Psychiatry.

[49] Bower J.E., Radin A., Ganz P.A., Irwin M.R., Cole S.W., Petersen L. (2025). Inflammation and dimensions of fatigue in women with early stage breast cancer: a longitudinal examination. Cancer.

[50] Ruiter A.M., van Meijgaarden K.E., Joosten S.A., Spitali P., Huijbers M.G., van Zwet E.W. (2025). Correlation of C-reactive protein with severe fatigue in patients with myasthenia gravis. Neurol Neuroimmunol Neuroinflamm.

[51] Lim W., Thomas K.S., Bardwell W.A., Dimsdale J.E. (2008). Which measures of obesity are related to depressive symptoms and in whom?. Psychosomatics.

[52] Resnick H.E., Carter E.A., Aloia M., Phillips B. (2006). Cross-sectional relationship of reported fatigue to obesity, diet, and physical activity: results from the third national health and nutrition examination survey. J Clin Sleep Med.

[53] Theorell-Haglöw J., Lindberg E., Janson C. (2006). What are the important risk factors for daytime sleepiness and fatigue in women?. Sleep.

[54] Vgontzas A.N., Bixler E.O., Chrousos G.P. (2006). Obesity-related sleepiness and fatigue: the role of the stress system and cytokines. Ann N Y Acad Sci.

[55] Zhang Q., Yun Y., An H., Zhao W., Ma T., Wang Z. (2022). Gut microbiome and daytime function in Chinese patients with major depressive disorder. J Psychosom Res.

[56] Coello K., Hansen T.H., Sørensen N., Munkholm K., Kessing L.V., Pedersen O. (2019). Gut microbiota composition in patients with newly diagnosed bipolar disorder and their unaffected first-degree relatives. Brain Behav Immun.

[57] Gupta A., Dhakan D.B., Maji A., Saxena R., Vishnu Prasoodanan P.K., Mahajan S. (2019). Association of Flavonifractor plautii, a flavonoid-degrading bacterium, with the gut microbiome of colorectal cancer patients in India. mSystems.

[58] Machiels K., Joossens M., Sabino J., Preter V.D., Arijs I., Eeckhaut V. (2014). A decrease of the butyrate-producing species Roseburia hominis and Faecalibacterium prausnitzii defines dysbiosis in patients with ulcerative colitis. Gut.

[59] Yekani M., Baghi H.B., Vahed S.Z., Ghanbari H., Hosseinpur R., Azargun R. (2021). Tightly controlled response to oxidative stress; an important factor in the tolerance of *Bacteroides fragilis*. Res Microbiol.

[60] Kim I., Hacker E., Ferrans C.E., Horswill C., Park C., Kapella M. (2018). Evaluation of fatigability measurement: integrative review. Geriatr Nurs.

[61] Huang W., Hickson L.J., Eirin A., Kirkland J.L., Lerman L.O. (2022). Cellular senescence: the good, the bad and the unknown. Nat Rev Nephrol.

[62] Riessland M., Orr M.E. (2023). Translating the biology of aging into new therapeutics for Alzheimer’s disease: senolytics. J Prev Alzheimers Dis.

[63] Zhang Y., Chi X., Zhou Q., Huang S., Chen H.-Z., Rosenzweig A. (2025). Cellular senescence and inter-organ communication in health and disease. Physiology (Bethesda).

[64] Ogata Y., Yamada T., Fujimura M., Igarashi T., Hasegawa S. (2024). Analysis of the relationship between age-related erythrocyte dysfunction and fatigue. Biogerontology.

[65] Guerville F., De Souto Barreto P., Ader I., Andrieu S., Casteilla L., Dray C. (2020). Revisiting the hallmarks of aging to identify markers of biological age. J Prev Alzheimers Dis.

[66] Soendenbroe C., Dahl C.L., Meulengracht C., Tamáš M., Svensson R.B., Schjerling P. (2022). Preserved stem cell content and innervation profile of elderly human skeletal muscle with lifelong recreational exercise. J Physiol.

[67] Luo D., Shang Z., He Q., Ke J., Xian Q., Dai S. (2025). The efficacy of resveratrol in the treatment of liver fibrosis: a systematic review and meta-analysis of preclinical studies. Front Nutr.

[68] Alway S.E., McCrory J.L., Kearcher K., Vickers A., Frear B., Gilleland D.L. (2017). Resveratrol enhances exercise-induced cellular and functional adaptations of skeletal muscle in older men and women. J Gerontol A Biol Sci Med Sci.

[69] Arnold P., Njemini R., Vantieghem S., Duchateau J., Mets T., Beyer I. (2017). Peripheral muscle fatigue in hospitalised geriatric patients is associated with circulating markers of inflammation. Exp Gerontol.

[70] Sorensen J.R., Skousen C., Holland A., Williams K., Hyldahl R.D. (2018). Acute extracellular matrix, inflammatory and MAPK response to lengthening contractions in elderly human skeletal muscle. Exp Gerontol.

[71] Bautmans I., Njemini R., Lambert M., Demanet C., Mets T. (2005). Circulating acute phase mediators and skeletal muscle performance in hospitalized geriatric patients. J Gerontol A Biol Sci Med Sci.

[72] Roerink M.E., van der Schaaf M.E., Dinarello C.A., Knoop H., van der Meer J.W.M. (2017). Interleukin-1 as a mediator of fatigue in disease: a narrative review. J Neuroinflammation.

[73] Latimer K.M., Gunther A., Kopec M. (2023).

[74] Xu J., Zhang R., Millischer V., Stiernborg M., Tume C.E., Mehdinia S. (2025). Elevated plasma GDF15 combined with FGF21 suggests mitochondrial dysfunction in a subgroup of anorexia nervosa patients. Transl Psychiatry.

[75] Lu W.-H., Sánchez-Sánchez J.L., de S. Barreto P. (2025). Appetite loss as a clinical marker of loss of function during ageing. Proc Nutr Soc.

[76] Huang Q., Trumpff C., Monzel A.S., Rausser S., Devine J., Liu C.C. (2025). The mitochondrial disease biomarker GDF15 is dynamic, quantifiable in saliva, and correlates with disease severity. Mol Genet Metab.

[77] Zhu Y., Zhu Y., Li J., Shi N., Li W., Tang Y. (2025). Research progress on intrinsic capacity in older adults: concepts, epidemiology, assessments, influencing factors, adverse outcomes, and interventions. Glob Health Med.

[78] World Health Organization (2015). https://iris.who.int/handle/10665/186463.

[79] Gaussens L., González-Bautista E., Bonnefoy M., Briand M., Tavassoli N., De Souto Barreto P. (2023). Associations between vitality/nutrition and the other domains of intrinsic capacity based on data from the INSPIRE ICOPE-care program. Nutrients.

[80] Koivunen K., Palmberg L., Lunansky G., Kok A., Glynn N.W., Cooper R. (2025). The interplay between perceived fatigability, intrinsic capacity, and physical activity: network analysis in a British birth cohort study. J Gerontol A Biol Sci Med Sci.

[81] Keshavarz M., Xie K., Schaaf K., Bano D., Ehninger D. (2023). Targeting the “hallmarks of aging” to slow aging and treat age-related disease: fact or fiction?. Mol Psychiatry.

[82] de Souto Barreto P., Rolland Y., Ferrucci L., Arai H., Bischoff-Ferrari H., Duque G. (2023). Looking at frailty and intrinsic capacity through a Geroscience lens: the ICFSR & Geroscience Task Force. Nat Aging.

[83] Groarke J.D., Crawford J., Collins S.M., Lubaczewski S., Roeland E.J., Naito T. (2024). Ponsegromab for the treatment of cancer cachexia. N Engl J Med.

[84] Ho L.Y.W., Ng S.S.M. (2020). Non-pharmacological interventions for fatigue in older adults: a systematic review and meta-analysis. Age Ageing.

