# Supplementary material for: Fatigue, muscle fatigability, and the Hallmarks of Aging: a narrative review
Source: J Nutr Health Aging. 2026 Apr 9;30(6):100844. doi: 10.1016/j.jnha.2026.100844 (PMC13091828; doi:10.1016/j.jnha.2026.100844)

**Supplementary Material Table-S1 – Search strategy and eligibility criteria**

*Figure S1* illustrates the chosen keywords for fatigue, aging and each of the twelve hallmarks of aging used to identify relevant articles, along with the number of studies obtained from each combination. The eligibility flow diagram is presented in *Figure S2*.

### Figure S1. Search term string and number of results identified for each hallmark.

| **A. Fatigue-related terms** |
| --- |
| “Fatigue” [MeSH Terms] OR “Fatigue” OR “Fatigability” OR “Tiredness” |

AND

| **B. Aging** |
| --- |
| "Aging"[MeSH Terms] OR "Aging"[Tiab] OR “Age” |

AND

| **C. Studies in humans** |
| --- |
| (Humans[Filter]) |

AND

| **D. Keywords for each of the hallmarks of aging** | | |
| --- | --- | --- |
| Hallmark | Search details | Number of results (combined search ‘A’, ‘B’ and ‘C’) |
| Genomic Instability | "Genomic Instability"[MeSH Terms] OR "DNA sequence, unstable"[MeSH Terms] OR "DNA Repair"[MeSH Terms] OR "DNA Damage"[MeSH Terms] OR "genes, mitochondrial"[MeSH Terms] OR "genome instabilit*"[Text Word] OR "genomic instabilit*"[Text Word] OR "genome stabilit*"[Text Word] OR "genomic stabilit*"[Text Word] OR "genetic instabilit*"[Text Word] OR "genome damage*"[Text Word] OR "genomic damage*"[Text Word] OR "genetic damage*"[Text Word] OR "genetic lesion*"[Text Word] OR "DNA repair*"[Text Word] OR "DNA damage*"[Text Word] OR "DNA lesion*"[Text Word] OR "nuclear DNA"[Text Word] OR "mitochondrial DNA"[Text Word] OR "nuclear lamina"[Text Word] OR "micronucleus assay*"[Text Word] OR "cf-DNA"[Text Word] OR "mtDNA"[Text Word] | 86 |
| Telomere Attrition | "Telomere Shortening"[MeSH Terms] OR "Telomere Homeostasis"[MeSH Terms] OR "telomere length*"[Text Word] OR "telomere attrition"[Text Word] OR "telomere shorten*"[All Fields] OR "telomerase"[All Fields] OR "telomere dysfunction"[All Fields] | 23 |
| Epigenetic Alterations | "Epigenomics"[MeSH Terms] OR "DNA methylation"[MeSH Terms] OR "Histones"[MeSH Terms] OR "Chromatin Assembly and Disassembly"[MeSH Terms] OR "epigenetic*"[Text Word] OR "histone*"[Text Word] OR "sir2"[Text Word] OR "sirt3"[Text Word] OR "sirt6"[Text Word] OR "DNA methylation"[Text Word] OR "chromatin remodeling"[Text Word] OR "polycomb"[Text Word] OR "transcriptome"[Text Word] OR "miRNA*"[Text Word] OR "microrna*"[Text Word] OR "non coding rna*"[Text Word] OR "circular RNA"[Text Word] OR "epigenetic clock"[Text Word] | 102 |
| Loss Of Proteostasis | "Proteostasis"[MeSH Terms] OR "Proteostasis Deficiencies"[MeSH Terms:noexp] OR "Heat-Shock Proteins"[MeSH Terms] OR "Chaperone-Mediated Autophagy"[MeSH Terms] OR "heat shock protein*"[Text Word] OR "stress protein*"[Text Word] OR "heat shock protein*"[Text Word] OR "Proteostasis"[Text Word] OR "proteasome"[Text Word] OR "Hsp70"[Text Word] OR "Hsp72"[Text Word] OR "heat-shock"[Text Word] OR "S6K1"[Text Word] OR "unfolded protein response"[Text Word] OR "Chaperone-Mediated Autophagy"[Text Word] OR "heat-shock protein"[Text Word] OR "misfolded protein"[Text Word] OR "proteomic*"[Text Word] OR "protein homeostasis"[Text Word] OR "proteostasis deficienc*"[Text Word] OR "proteostasis dysfunction*"[Text Word] OR "Protein Misfolding"[Text Word] OR "Protein Folding"[Text Word] | 82 |
| Disabled Macro-autophagy | "Autophagy"[MeSH Terms] OR "Macroautophagy"[MeSH Terms] OR "macro autophagy"[Text Word] OR "macro autophagy"[Text Word] OR "Cellular Autophagy"[Text Word] OR "Autophagocytosis"[Text Word] | 3 |
| Deregulated Nutrient Sensing | “nutrient sens*"[Text Word] OR "insulin"[Text Word] OR "IGF1"[Text Word] OR "insulin-like growth factor"[Text Word] OR "GH"[Text Word] OR "growth hormone"[Text Word] OR "IIS"[Text Word] OR "AKT"[Text Word] OR "FOXO"[Text Word] OR "PI3K"[Text Word] OR "mTOR"[Text Word] OR "SIRT"[Text Word] OR "sirtuin*"[Text Word] OR "sirt1"[Text Word] OR "AMPK"[Text Word] OR "dietetic restriction"[Text Word] OR "fasting"[Text Word] OR "somatotropic axis"[Text Word] OR "metabolic pathway"[Text Word] | 514 |
| Mitochondrial Dysfunction | "Mitochondria"[MeSH Terms] OR "mitochondrial dysfunction*"[Text Word] OR "mitochondrial integrity"[Text Word] OR "mitochondrial decline"[Text Word] OR "reactive oxygen species"[Text Word] OR "ROS"[Text Word] OR "PGC-1"[Text Word] OR "apelin"[Text Word] | 140 |
| Cellular Senescence | “Cellular Senescence"[MeSH Terms:noexp] OR "senescence associated secretory phenotype"[MeSH Terms] OR "senescence associated secretory phenotype"[Text Word] OR "SASP"[Text Word] OR "cell senescence"[Text Word] OR "cell aging"[Text Word] OR "cell ageing"[Text Word] OR "senescent cell*"[Text Word] OR "P16INK4A"[Text Word] OR "p19ARF"[Text Word] OR "INK4a/ARF"[Text Word] OR "senescence-associated beta-galactosidase"[Text Word] OR "p53"[Text Word] OR "senolytic*"[Text Word] | 35 |
| Stem Cell Exhaustion | "Stem Cells"[MeSH Terms] OR "Cellular Reprogramming"[MeSH Terms] OR "tissue renovation"[Text Word] OR "cell reprogramming"[Text Word] OR "stem cell*"[Text Word] OR "progenitor cell*"[Text Word] OR "stem cell exhaustion"[Text Word] OR "stem cell aging"[Text Word] OR "stem cell decline"[Text Word] OR "hematopoietic stem cell*"[Text Word] OR "HSCs"[Text Word] OR "circulating osteogenic progenitor"[Text Word] OR "circulating osteoprogenitor"[Text Word] OR "circulating osteogenic precursor"[Text Word] | 230 |
| Altered Intercellular Communication | "Cell Communication"[MeSH Terms] OR "Paracrine Communication"[MeSH Terms] OR "Oxidative Stress"[MeSH Terms] OR "Reactive Oxygen Species"[MeSH Terms] OR "Lymphotoxin-alpha"[MeSH Terms] OR "Interleukin-6"[MeSH Terms] OR "Chemokine CCL11"[MeSH Terms] OR "Extracellular Matrix"[MeSH Terms] OR "Nitric Oxide"[MeSH Terms] OR "intercellular communication"[Text Word] OR "Cell Communication"[Text Word] OR "Reactive Oxygen Species"[Text Word] OR "ROS"[Text Word] OR "CCL11"[Text Word] OR "IL6"[Text Word] OR "Interleukin-6"[Text Word] OR "TNFB"[Text Word] OR "Tumor Necrosis Factor beta"[Text Word] OR "TNF-beta"[Text Word] OR "extracellular matr*"[Text Word] OR "fibroaging"[Text Word] OR "Nitric Oxide"[Text Word] OR "Oxidative Stress"[Text Word] | 461 |
| Chronic Inflammation | "Inflammation"[MeSH Terms:noexp] OR "C-Reactive Protein"[MeSH Terms] OR "tumor necrosis factor alpha"[MeSH Terms] OR "inflammat*"[Text Word] OR "inflammaging"[Text Word] OR "inflammasome*"[Text Word] OR "inflammatory cytokine*"[Text Word] OR "pro inflammatory cytokine*"[Text Word] OR "NF-kappaB"[Text Word] OR "NLRP3"[Text Word] OR "GnRH"[Text Word] OR "sirt2"[Text Word] OR "CD4 T"[Text Word] OR "IL-6"[Text Word] OR "Interleukin-6"[Text Word] OR "TNFalpha"[Text Word] OR "tumor necrosis factor alpha"[Text Word] | 1604 |
| Dysbiosis | "Dysbiosis"[MeSH Terms] OR "Microbiota"[MeSH Terms] OR "Gastrointestinal Microbiome"[MeSH Terms] OR "Dysbioses"[Text Word] OR "Disbiosis"[Text Word] OR "Disbioses"[Text Word] OR "microbiot*"[Text Word] OR "microbial communit*"[Text Word] OR "microbiom*"[Text Word] OR "gut microbiom*"[Text Word] OR "Gastrointestinal Flora"[Text Word] OR "Gut Microflora"[Text Word] OR "Enteric Bacteria"[Text Word] | 50 |

### Figure S2. Eligibility flow diagram


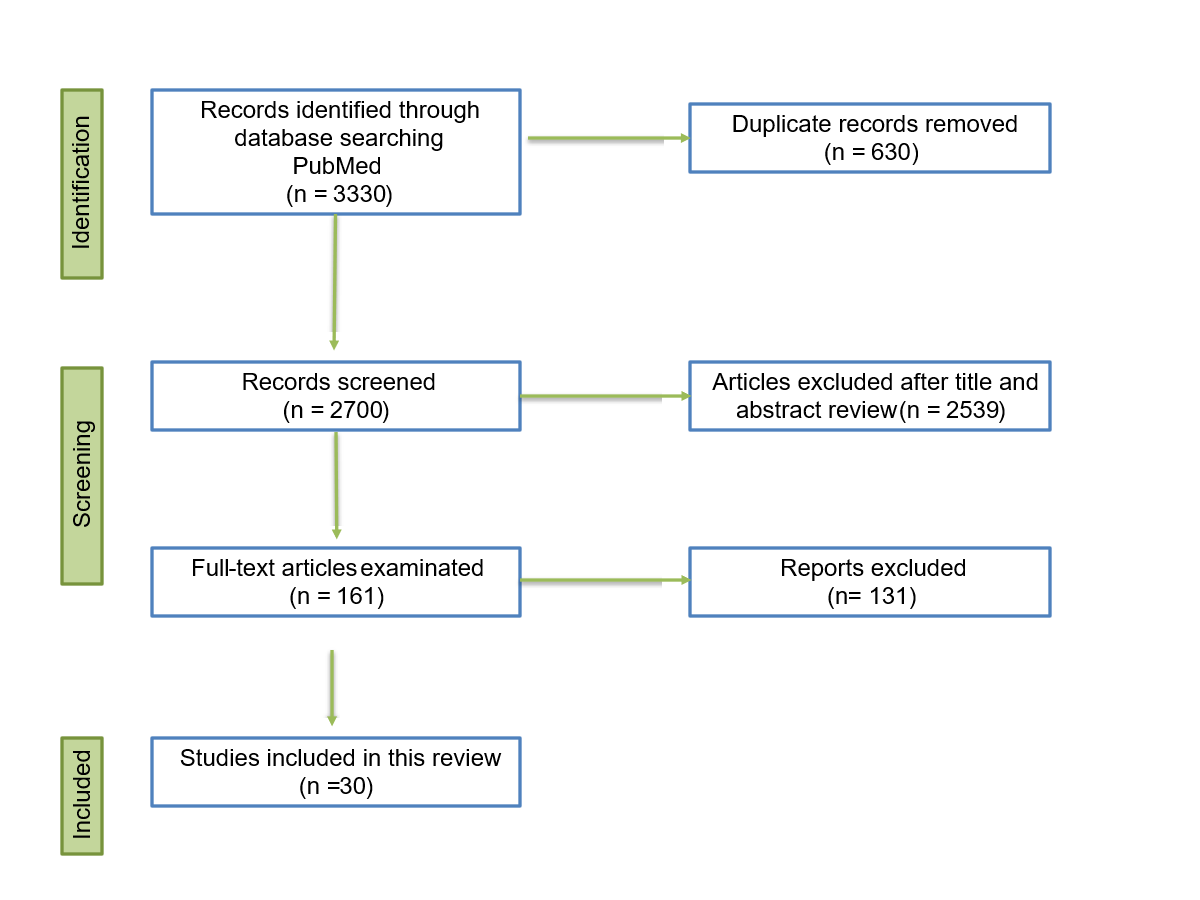

Supplement: Supplementary file 1 [file mmc1.docx]
